# Exploring Anatomic Variants to Enhance Anatomy Teaching: Musculus Sternalis

**DOI:** 10.3390/diagnostics10080508

**Published:** 2020-07-22

**Authors:** Andrew J. Petto, David E. Zimmerman, Elizabeth K. Johnson, Lucas Gauthier, James T. Menor, Nicholas Wohkittel

**Affiliations:** 1 Department of Biological Sciences and Integrative Health and Assessment Unit, Department of Kinesiology, University of Wisconsin, Milwaukee, WI 53201-0413, USA; 2 School of Medicine, St George’s University, University Centre, True Blue Bay, Grenada 262G 5G , West Indies; dzimmerm@sgu.edu; 3 Department of Social Sciences, Southern New Hampshire University, Manchester, NH 03106, USA; e.johnson4@snhu.edu; 4 Advanced Physical Therapy & Sports Medicine Clinic, Marinette, WI 54143, USA; lgauthi25@gmail.com; 5 Milwaukee Public Schools, Milwaukee, WI 53208, USA; menorjt@milwaukee.k12.wi.us; 6 Froedtert & The Medical College of Wisconsin, Wauwatosa, WI 53226, USA; nickwoahkittel@gmail.com

**Keywords:** M sternalis, anatomic variation, learning strategies

## Abstract

The opportunity to encounter and appreciate the range of human variation in anatomic structures—and its potential impact on related structures, function, and treatment—is one of the chief benefits of cadaveric dissection for students in clinical preprofessional programs. The dissection lab is also where students can examine unusual anatomic variants that may not be included in their textbooks, lab manuals, or other course materials. For students specializing in physical medicine, awareness and understanding of muscle variants has a practical relevance to their preparations for clinical practice. In a routine dissection of the superficial chest muscles, graduate students in a human gross anatomy class exposed a large, well-developed sternalis muscle. The exposure of this muscle generated many student questions about M sternalis: its prevalence and appearance, its function, its development, and its evolutionary roots. Students used an inquiry protocol to guide their searches through relevant literature to gather this information. Instructors developed a decision tree to assist students in their inquiries, both by helping them to make analytic inferences and by highlighting areas of interest needing further investigation. Answering these questions enriches the understanding and promotes “habits of mind” for exploring musculoskeletal anatomy beyond simple descriptions of function and structure.

## 1. Introduction

“No two bodies are exactly alike.” We repeat this adage in every anatomy class; but students rarely observe significant anatomic variation directly, unless they have access either to advanced imaging or cadaveric dissection. For students in clinical preprofessional programs, the direct comparison of sizes, shapes, proportions, and relationships among anatomic structures has several potential benefits that can enhance the appreciation—and often the clinical relevance—of anatomic variation in their career preparation.

Among the variants sometimes revealed in anatomic dissection, students oten encounter features that are rare, unexpected, or just “not supposed to be there”. Since there are literally thousands of such variants documented in the literature [1], it is not surprising that these should appear occasionally in routine anatomy instruction, such as in laboratory dissection sessions. When our students encounter these variants, there is a unique opportunity for instructors to help students see these structures as more than simple curiosities, and to place them instead into a broader context of influences that produce an anatomic form and that may have a practical relevance in clinical practice.

One major emphasis of our anatomy curriculum is to promote the understanding of anatomic structure and function as the result of the dynamic interplay of several biologic processes. Often, even in courses that focus on human movement, there is a tendency in textbooks and reference materials to treat the gross anatomy of the musculoskeletal system as invariant. Curricular presentations of musculoskeletal anatomy often involve little more than a summary of location, attachments, innervations, and typical actions. But, when students uncover unusual anatomic variations in the gross anatomy lab, there is an opportunity for instructors and students to use these examples to deepen the understanding and appreciation of how the musculoskeletal system is formed and attains its usual presentation.

In this paper, we demonstrate this approach, taking advantage of the opportunity presented by an unusual anatomic feature―a large, unilateral M sternalis―to illuminate the various biologic processes that contribute to the appearance and function of the musculoskeletal components of adult human anatomy. This report is focused on the application of a pedagogic approach to learning anatomy: a problem-based exploration that guides students through the functional, developmental, and phylogenetic influences on musculoskeletal form and function. The object is to help students develop “habits of mind” based on a process of disciplined inquiry that provides a framework for understanding anatomic features that can also be applied to help students interpret the significance of anomalous or unusual musculoskeletal variants in the context of how the processes that produce the usual anatomic features can also produce the unusual.

In our human gross anatomy course for doctoral students in physical therapy (KIN525: Human Gross Anatomy), one group of students carrying out routine dissection of the thorax exposed a well-developed and relatively large M sternalis, which they discovered was not included in the reference materials and dissection guides used in the course. This discovery prompted both a high level of excitement and an opportunity to use this excitement to promote a deeper understanding of adult musculoskeletal morphology.

When an unusual variant appears in the anatomy lab, the first questions from students are “What is it?” and “Where did this come from?” In this report, we will use this example of students’ isolation of an unusually well-defined M sternalis as an example for how anatomic variation can be a gateway to deeper learning about the variables that affect the anatomic features that they will encounter professionally. Answering students’ questions about the appearance of this muscle helped us to explore both the usual anatomic features that they encounter in the human body and the unusual variants that present themselves on occasion.

The first place students look for answers, of course, is in their anatomic atlases and dissection guide. There are a few additional resources that contain detailed observations about anatomic variations in human anatomy [1,2,3,4]. Bergman et al. [1,2] have one of the most comprehensive resources for anatomic variations in humans, illustrating an impressive wealth of these variants in multiple organs and systems. Platzer is somewhat more accessible in that its descriptions of variants are included in the same parts of the text as related structures in the musculoskeletal, nervous, and vascular systems [4]. Diogo and Abdala combine a detailed search of descriptions of anatomy literature for humans and their primate relatives supplemented with Diogo’s own careful dissection of relevant specimens [5]. Diogo and Wood’s comprehensive volume contains a description of M sternalis in humans and other apes [3].

The initial review of available literature on M sternalis suggested that the appearance of this muscle is a “rare” variant (though what constitutes “rare” is seldom quantified). Its typical prevalence is estimated at 3‒5% of individuals [6,7], but other sources estimate higher rates of up to 20% based on a literature review [8]. It is of note that Jelev et al. estimated that prevalence rates in European populations averaged 4.7% but that prevalence was higher in African (10%) and Asian (up to 20%) populations [8]. A systematic survey in a Chinese population (estimates prevalence rates around 6% [9]). These latter studies suggest some variations in prevalence among regional geographic populations, and the lower rates in the earlier reports may result from the tendency to collect data fromsubjects of western European ancestry. Eisler confirms this with his survey of reports from Europe, where reported prevalence was under 10% and in the Far East, where prevalence was up to 15% [10].

In modern times, M sternalis may be encountered in thoracic surgery or in diagnostic imaging, such as mammography [11,12,13], which may account for a slightly higher reported prevalence for this muscle in females than in males. However, most reports of this anatomic variant arise from serendipitous findings, like the one in our teaching lab.

The earliest report in the western anatomic literature appears to be from a series of short observations on anatomic variations in Cabrolio [14]. Turner [6] credits Cochon-DuPuy [15] with the earliest attempt to describe M sternalis in relation to the other superficial anterior muscles—in this case in association with Rectus abdominis. Most 19th century sources point to the work of Albinus as the source for the first systematic and detailed description of this muscle [16]. Albinus [16] describes this variant as a rare example of nature’s playfulness (or perhaps trickery or mockery): “*rarum naturae ludentis exemplum*.” The earliest report in English appears to be M’Whinnie whose review suggested that M sternalis (which he referred to as Rectus sternalis) was commonly considered to be associated with either Rectus abdominis or M sternocleidomastoideus because of its location, topography, and most common attachments [17].

Turner appears to be the first to examine enough cadavers to estimate a prevalence (21 of 651, or about 3%), and he noted several different arrangements of the muscle [6]. He also reviewed the literature available to him at that time and reported that variants of this muscle were named in the literature as M sternalis, M presternalis, M rectus sternalis, M sternalis brutorum, or M thoracicus [6,18].

**Figure 1:**
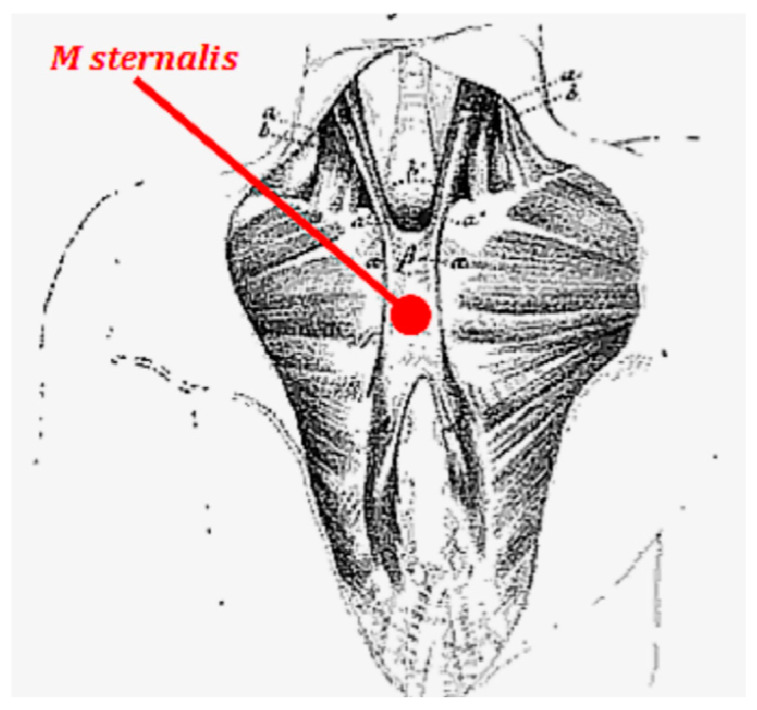
A bilateral M sternalis; the contralateral clavicular attachment is characteristic of Type II-1 in Jelev et al. [8]. Source: Figure 3 in Gruber [19]. Public domain based on publication date.

Most sources describe a strap-like muscle parallel and slightly lateral to the sternum and superficial to the pectoralis muscles. The muscle may be truly bilateral (with right and left muscles about equal in length and mass, and having similar or identical mirror-image attachments). However, Jelev et al. illustrate eight different arrangements with many variations based on the locations of the muscle bodies and their attachments [8]. Four variants are classified as Type I with attachments on the lower ribs only on one side of the chest, even if the clavicular attachments are bilateral. The four Type II variants all have attachments on the lower ribs on both sides of the chest, often with a crossing over to attach to the contralateral clavicle (Figure 1; Gruber [19]).

Although there seems to be general agreement on the location, attachments, and topographic associations of M sternalis in the literature, despite its anatomic variability, its innervation remains a matter of disagreement. But even case reports based on dissection of the muscle and its neurovascular supply have different findings.

Some report innervation only from intercostal nerves. Sarikçioğlu et al. identified the anterior cutaneous branch of intercostal nerve 6 as supplying the M sternalis muscle [20]. Natsis and Totlis and Hung et al. both identify the innervation as from a branch of intercostal nerve 2 [21,22]. Arráez-Aybar et al. report a “neurovascular pedicle” in the mid-portion of the M sternalis arising from the anterior cutaneous branch of intercostal nerve 3 (and supplied by anterior intercostal arteries from the internal thoracic artery and vein) [23].

Other sources identify innervation from the anterior or medial thoracic nerves. Katara et al. identify “twigs” of the pectoral nerve, but do not identify which [24]. Snosek et al. report that innervation is from the medial pectoral nerve [13]. Kida et al. are adamant that the only innervation is from the medial pectoral nerve [25]. And others, as far back as Eisler [10], suggest that the branches of the intercostal nerves that are seen in association with the M sternalis penetrate the muscle on their way to innervate the overlying skin or other nearby tissues, but do not serve the muscle itself [13,25,26].

To complicate things more, Pillay et al. report the innervation of a unilateral M sternalis via the medial pectoral nerve, but that of a bilateral M sternalis by intercostal nerves [27]. And Hung et al., also report finding innervation from both the intercostal and the medial pectoral nerves [22]. Among those reviewing the literature, both Vaithianathan et al. and Raikos et al. cite a report by O’Neil and Folan-Curran that 55% of cases indicate innervation by a pectoral nerve, 43% indicate innervation by intercostal nerves, and 2% indicate innervation by both [28,29,30].

Raikos suggests that the variability in innervation may have a developmental aspect [28]. The pattern of innervation may represent opportunistic connections between myocytes and neurons based on the topographic location of the precursor to M sternalis. There is also a significant number of reports of the appearance of M sternalis in anencephalic fetuses and infants [7,13,22,28,31]. However, the example we encountered occurs in an otherwise anatomically normal adult female, so major developmental anomalies appear to have no bearing on the appearance of this muscle in our case.

## 2. Initial Observation

**Figure 2:**
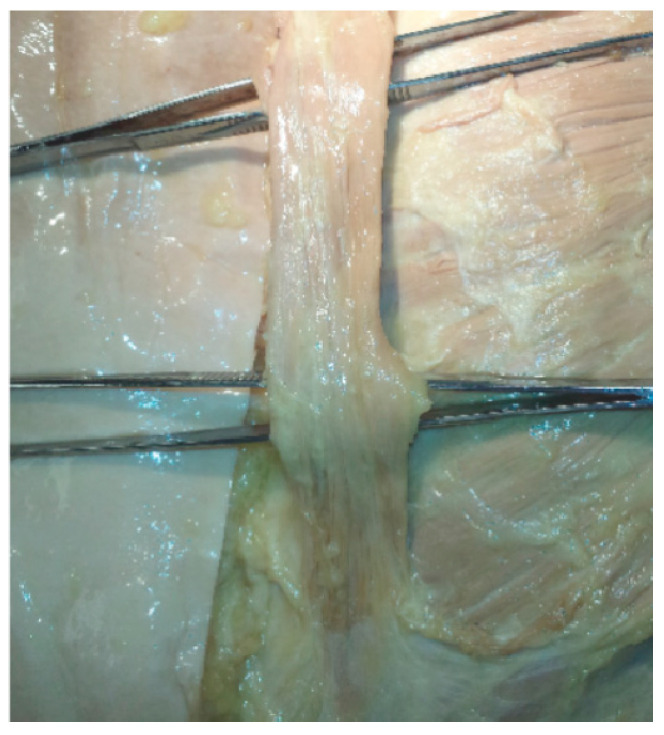
Photograph of M sternalis exposed during routine dissection in human gross anatomy laboratory; 25 cm forceps were used to indicate scale. Photograph by AJ Petto.

Doctoral students in the physical therapy program at the University of Wisconsin‒Milwaukee (UWM) uncovered a superficial band of muscle overlying the left lateral edge of the sternum and adjacent costochondral cartilages lying between the subcutaneous fascia and the pectoral muscles in an 87-year-old female during a routine dissection of the chest (Figure 2). A flat band of parallel fascicles was connected by a merger of the fascia cranially into connective tissues associated with the sternoclavicular joint. There was a similar merging between the fascia at the caudal extent of the muscle and the fascia associated with the M rectus abdominis. This muscle extended 18 cm from near the xiphoid process to the sternoclavicular joint and ranged from 1.5 to about 2 cm wide. There were no other unusual features uncovered in the dissection, including in the thorax, the remainder of the musculoskeletal dissection, or in the gross appearance of organs in the chest and abdomen.

The original dissection did not preserve all the contributing nerves or blood vessels, but the initial appearance was that the muscle was served by several nerves and vessels attaching at intervals along its length that emanated from the anterior chest wall. The impression was of nerves emerging from the intercostal spaces, but the individual nerves were not traced back to their spinal roots. The removal of overlying tissues before discovery of the M sternalis in this donor also made it impossible to verify the suggestions generated later by the literature review that these nerves might penetrate the muscle to serve the fascia and skin above the muscle layer.

## 3. The Essential Questions

For any anatomic structure—perhaps especially for unusual variants—we want to learn why it appears as it does, if it has any function, and what its source is. We begin by exploring the typical explanations for such structural variation: Vestiges (or vestigial structures); anomalies (unusual development); remnants or rudimentary muscles (incomplete development via myogenesis or apoptosis), or atavisms (or “throwbacks”). These are described in more detail in Table 1.

By exploring each of these potential explanations for the appearance of M sternalis in this donor, students can ultimately learn more about muscle function and development in general. Then they can apply this knowledge to muscle anatomy in other cases—for both the unusual and the expected anatomic presentation of skeletal muscles. In our labs, we use a mnemonic for engaging a deeper understanding of gross anatomic features: F⦁E⦁D⦁U⦁P (Function‒Evolution‒Development Understanding Protocol). This “protocol” guides student inquiry as they seek to learn more about specific aspects of any anatomic feature, but particularly in their exploration of information about unusual variants.

Function is the component of the mnemonic that is easiest for students to grasp. Function refers to the outcome of muscle action: when this muscle contracts, what are the effects on movement, position, or posture? Does the muscle’s action cause or resist movement of any segment of the body? Does it result in or prevent a change in the relationship of the main axis of the body to the substrate (position)? Does it result in or resist a change in posture? These aspects of musculoskeletal function are typically available in most standard anatomy texts.

Evolution is the component of the mnemonic that most students have not considered―at least in the context of their studies in anatomy. Even those students who have experience in comparative anatomy—at a minimum, those who have dissected fetal pigs, cats, or other vertebrates in their anatomy or biology courses—tend not to think in evolutionary terms about the similarities and differences.

Evolution traces the peculiar combination of anatomic features that define the branching patterns in the tree of life. Branches are defined by the emergence of derived states of anatomic features that are shared by a group of descendants and their common ancestors but that separate them from organisms on other branches (for example, the lack of an external tail among all apes and their descendants; [34]). For students of human anatomy, the *phylogenetic* pattern illustrates the history of anatomic changes that the earliest humans inherited from populations of their ancestors and that are used as a basis for the anatomic specializations that define our species. Zanni and Opitz lay out a general approach to analyzing the evolutionary components of usual and unusual anatomic features [35].

Development is the third component, and, though students are aware of this aspect, they often have not given much thought specifically to how developmental processes result in the anatomic form they see in their dissections. Often our students are most interested in muscle conditioning and body building, and somewhat less in apoptosis and myogenesis, than in embryologic processes for muscle differentiation, migration, and attachment. Understanding how muscles come to be in their typical locations, with their typical sizes, shapes, attachments, innervation, and vascular supply is usually only challenged and brought to the forefront when a student uncovers a muscle with unexpected characteristics.

All these components together help us to resolve the essential questions and classify unexpected variants in the anatomy lab as indicated in Table 1. With the answers that result from asking about function, evolution, and development, students can use a graphic organizer—described in the discussion section—to apply the information they have uncovered to an anatomic variant. Students follow steps in a “decision tree” to a resolution of the essential questions or to indicate specific information based on direct observation of the dissection and from the literature review conducted in the process of applying the F⦁E⦁D⦁U⦁P protocol that students still need to locate before a step in the decision tree can be completed. The literature reported in the following sections reflects how students progress through the protocol. The goal is to locate relevant sources that address all three aspects of the protocol as they relate to observed anatomic features and to understand their contributions to the anatomic form they have observed.

### 3.1. Function

After answering the first question on exposing M sternalis—“What is it?”—the next question is often, “What does it do?” More specifically, when the muscle is activated and develops tension, is there any change in the position of skeletal elements located between its attachments? Students in our gross anatomy lab apply a standard template when studying all skeletal muscles to relate the function of a muscle with its location and attachments (See Table 2), and this can be applied to any muscle that presents in their dissections.

Students in the Human Gross Anatomy course take their findings from this template to a concurrent course, Introduction to Physical Therapy Practice Examination Techniques (KIN526). In this concurrent course, they practice locating musculoskeletal structures by physical examination and consider the clinical implications of any atypical findings.

Generally speaking, it is easier to answer this question when the appearance of a muscle is regular and consistent from one individual to the next. However, the reports of the location, shape, and attachments of M sternalis indicate that there is considerable variability in its morphology [8,28].

We expect that a muscle that appears highly variable anatomically would be less likely to have any essential function. This is not to say that the muscle could never have any effect on the skeletal elements located between its attachments, but rather that such an effect might be idiosyncratic—dependent on the specific muscle morphology, and not generalizable in a way that would apply to all the ways that the muscle can appear. Any regular action that would affect the positions of skeletal elements engaged by the muscle body would probably rely on other, more regular muscles as their primary movers.

**Figure 3:**
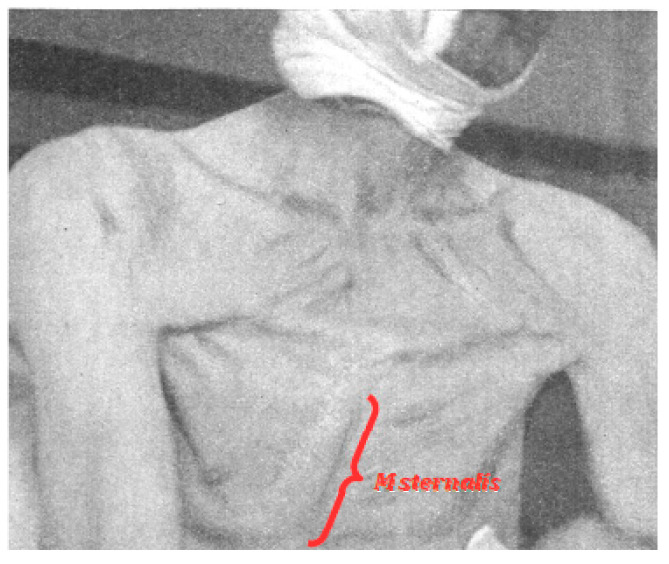
Image from Kirk showing tension in a unilateral, right M sternalis [36]. Image in the public domain based on publication date.

Until recently, examples of M sternalis have been reported almost entirely from studies of cadavers, so function could be inferred, but not confirmed. One exception comes from Kirk who was able to show the surface definition of the muscle under tension [36] (Figure 3). The author produced this effect “with the recti abdominis in flexion of the trunk, and, as is shown, with the pectorales in adduction of the arms; and its origin could then be seen to spread out transversely over the lower part of the Pectoralis major”.

This description does not provide a clear answer on specific function, since the postural changes include both the adduction of the left arm by the Pectoralis major and the flexion of the trunk by the Rectus abdominis. However, it does give support to the two major candidates for the source of this muscle: P major and R abdominis, even if it does not resolve the question in favor of one or the other [13].

### 3.2. Evolution

The search for the evolutionary foundations of human morphology begins in comparative anatomy. Table 3 provides a summary of the search through the anatomic literature for the appearance of chest muscles in addition to the muscles of the pectoral girdle or the intercostals. In Urodeles, Hildebrand shows a continuous ventral muscle body from the pubis to the cephalad border of the sternum: a homolog of the R abdominis [37]. Omura et al. confirm that the Rectus group is active in maintaining posture on land in resistance to vertebral bending under gravity, thus their main function does not appear to involve ventilation of the lungs [38].

In mammals, Getty reports a Rectus thoracis muscle in ruminants and horses that lies over the ventral chest wall and extends from the cephalad aspect of the R abdominis to the top of the sternum [39]. In these animals, Getty reports that the muscle appears to assist in expansion of the chest cavity under conditions of aggressive inhalation, such as when running [39].

Among the primates, there are two patterns of ventral muscles worth noting. First, the R abdominis generally tends to attach more cephalad than is typical in humans [35,36], often on the manubrium [40,43,44] or as high as the first rib [44]. The naming of additional muscle bodies that are described as cephalad extensions of the aponeurosis of the R abdominis varies to include the names found in the older literature (discussed above). Osman Hill refers to this additional muscle as Rectus sternalis, while Diogo and Wood, only in hominids, refer to the muscle as simply M sternalis as found in *Homo* and *Hylobates syndactylus* (the siamang) [3,42].

Turner points out that part of the confusion about the attachments of R abdominis may derive from the anatomic work of Galen who used dissections of nonhuman primates as the basis for at least some of his descriptions of human anatomy [6]. Therefore, Turner argued, early anatomists were misled about the cephalad attachment of the R abdominis in normal human cadavers [6]. If this is the case, these anatomists might conclude that the appearance of an additional superficial strap-like ventral muscle extending to the sternum or clavicle might simply represent an elongation of or variation in the R abdominis.

Another candidate suggested as the basis for M sternalis is the Panniculus carnosus [6,47,48]. In other mammals, this muscle is typically located below the adipose layer lying deep to the dermis and above a connective tissue layer that separates the integument from the underlying skeletal muscle layer [48]; but it does not have any direct skeletal attachments [47]. Generally speaking, P carnosus does not appear in the higher primates (including hominins) as a distinct muscle of the trunk. However, Langworthy argues that this muscle is derived from the pectoral group in mammals that retain it, and cites Eisler’s (1912) argument that M sternalis is formed by a failure of proper development in the pectoral musculature [10,47]. Bergman et al. also suggest that remnants of P carnosus may be found in the pars abdominalis of the pectoralis muscles or as “extra, independent, muscular slips from the abdominal aponeurosis which spreads forward on the rectus sheath” (https://www.anatomyatlases.org/AnatomicVariants/MuscularSystem/Text/P/05Panniculus.shtml) [1].

Naldaiz-Gastesi et al. identify 12 other muscles or muscular structures of the trunk and neck as potentially derived from, or containing, remnants of P carnosus, the most obvious of which is the Platysma [48]. These remnants, they argue, perform many functions—at least in nonhuman species—and some are incorporated into other regular, named muscles in the trunk. Based on their comparisons of the form and function of P carnosus in several mammalian species, Naldaiz-Gastesi et al. conclude that the innervation of P carnosus is independent of that of the underlying skeletal muscles allowing it to function separately from these other muscles [48]. If P carnosus is the source of M sternalis, then this difference in innervation might be consistent with the lack of apparent movement of skeletal elements by the M sternalis noted in the human anatomy literature.

However, these similarities do not by themselves help us to resolve the question of the evolutionary foundations of the appearance of M sternalis. Once we have described the patterns of similarities and differences in related organisms, we use these patterns to construct a cladogram or phylogenetic tree to establish the pattern of “descent with modification” that an evolutionary analysis requires [5]. In general, we are looking for an anatomic change that is established in an ancestral population and is shared by all the descendants of that population. It is also possible to define a group by a shared absence of a feature common in all its evolutionary relatives, because its ancestors have modified or eliminated it.

For example, a post-anal tail is considered a shared, conservative trait for the vertebrates, but none of the hominids (humans and other apes) retain this feature. The *loss* of the feature is a shared derived trait that helps to define the organisms on the ape evolutionary branch from those on other primate branches [34]. However, the loss of tail in some of the other primates or in other non-primate vertebrate species is understood as a repeatedly derived trait, that is, a feature that appears similar in several species but does not arise by common descent [49].

**Figure 4:**
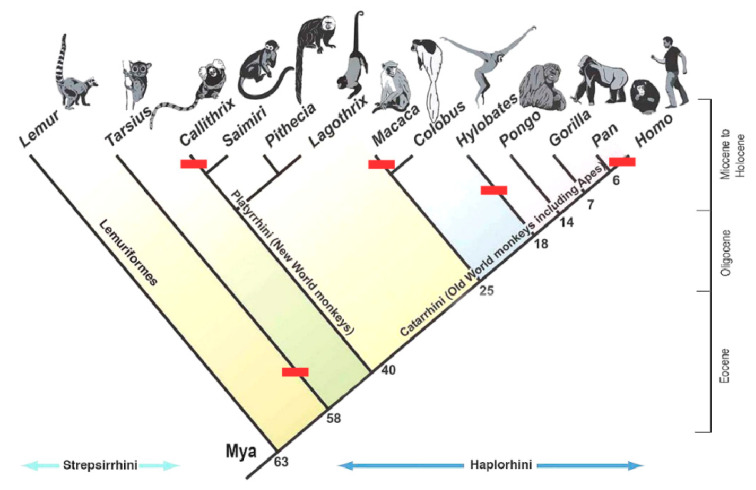
Cladogram showing the appearance and pattern of phylogenetic inheritance for muscles identified as potentially homologous with M sternalis in humans. Red bars indicate the branch on which the character state (presence of M sternalis or homologous muscle) appears. Data used for the character states described in Table 3. Image source: Available under Creative Commons Licensing from https://upload.wikimedia.org/wikipedia/en/thumb/f/f0/PrimateTree2.jpg/1280px-PrimateTree2.jpg.

When we superimpose the patterns of ventral muscular anatomy on the primate cladogram (Figure 4), it is clear that M sternalis fails the test of a shared derived trait among the primates or even the hominids. When this muscle—or other muscles that may or may not be the same—appears in our phylogenetic diagram, it follows the pattern of a repeatedly derived trait: one that arises only on side branches unique to specific taxa, rather than at locations in the cladogram that join several lineages together (nodes) by virtue of their sharing this feature by way of descent from a common ancestor.

### 3.3. Development

Our students do not typically study the details of the developmental processes that produce the muscles they encounter in the gross anatomy lab. For most of them, any background in developmental biology is limited to sections in their introductory biology or anatomy-and-physiology texts, or from a general overview, such as is found in Wolpert [50]. As a result, they are aware of the basics: muscles form from the dorsolateral aspects of the somites [51], transcription factors cause cellular differentiation, coalescence into muscle tissue is mediated by adhesion molecules (though it is possible for cells in these early tissues to dissociate and re-associate with other cells) and the final shape of the tissue can change in this process, *Hox* genes provide positional information, but developing muscles can be influenced by external conditions: the epigenetic influences from extracellular components and the availability of attachment sites in the underlying morphology [50]. Wolpert describes the ultimate musculoskeletal attachments as “democratic” and elsewhere as “promiscuous” in that these tissues and their associated connective tissues will attach to any appropriate substrate in their vicinity [50].

Understanding the contingent nature of much of developmental biology is perhaps the single greatest challenge for how students view developmental processes at this early stage of morphogenesis and their influences on adult anatomy. They need to develop the appreciation that development does not follow strict “blueprints” so much as a general schematic for the final form and location of skeletal muscles, and that the final result can be affected by numerous influences along the way.

Perhaps the most useful approach for students in the gross anatomy lab is to partition developmental processes into the effects whose results are more readily observable in the adult cadaver they encounter in the gross anatomy lab. These would look for evidence of (1) differentiation into muscle tissue from myocytes; (2) migration of muscle tissue to proper locations; and (3) formation of attachments appropriate for normal function.

#### 3.3.1. Differentiation

Postcranial musculoskeletal development is remarkably conserved in vertebrates, such that processes relevant to human development can be observed in different model organisms [52]. Differentiation of the cells destined to be muscles are influenced by a muscle transcription factor (*MyoD*) to produce myoblasts [51]. Several myogenic regulatory factors are involved in the specification and differentiation of muscle tissues, and Pownall et al. detail multiple influences on myogenesis at different locations in the embryo [53].

According to Shearman and Burke, myoblasts are not committed a priori to specific muscles, but the connective tissues with which the myoblasts associate will dictate their final destinations [52]. These associations produce muscle bundles that retain their segmentation in the thoracic region, while the ventral portion of the bundles that will become the abdominal muscles fuse into the Rectus abdominis [51]. The presomitic mesoderm that will populate the thorax produces the connective tissue template for thoracic muscles regardless of where they are finally located [52]. Pownall et al. confirm that ectopic development of muscle masses in experimental studies is rare; that is, how a muscle develops depends a great deal on where its precursors are located [53].

Mekonen et al. indicate that the musculoskeletal primordia of the thorax appear as recognizable tissues by 5.5 weeks in the human embryo, and the establishment of the abdominal muscles is complete by week 10 [54]. For the M sternalis, a problem in this part of the process should be evident in malformation or other defects of the muscle tissue [54].

#### 3.3.2. Migration

Typically, the development of bone, tendon, and muscle that will form a functional unit is coordinated to produce a functional whole [55]. Pownall et al. [53] report that there is a relatively small mass of migratory cells for populating the embryonic body wall and limbs, and that a myogenic factor (*MyoD*) is responsible for differentiation of muscle masses “in coordination with tendon and bone formation”.

In several documented examples of problems in pectoral muscle development, underlying malformations in the connective tissues associated with skeletal attachments are common, for example in Poland syndrome (https://omim.org/entry/173800). In a case study of atypical muscle formation associated with Mm pectorales, Bannur et al. review atypical formations of pectoral musculature and suggest that the locations of muscle attachments can be useful in tracing their developmental histories in the interplay between migration, fusion, and apoptosis [56].

The atypical appearance of these muscle variants is often associated with at least one uncharacteristic attachment. This condition is consistent with Wolpert’s characterization of muscle attachment as “democratic”, in which skeletal muscles form attachments to connective tissue structures in nearby locations, rather than searching for fixed, pre-set attachment points [50]. For the M sternalis, a problem in this part of the process should be evident in atypical formations of connective tissues associated with the muscle.

#### 3.3.3. Attachments

Early muscle development involves activation of myogenic factors, differentiation of myoblasts, and proliferation of myocytes. However, as Hasson reports, the final association between these muscle masses and specific skeletal muscles is not predetermined [57]. The role of the connective tissues within and surrounding the developing muscles helps to determine the patterning of these muscles that will result in the gross muscle anatomy we see in dissection.

Hasson (2011) reviews experimental studies on how the patterning in developing skeletal muscle arises and how the associations between the muscles and the related skeletal elements may be uncoupled. In our case of M sternalis, at least one attachment can be associated with those proposed as the ultimate source of the muscle (Rectus abdominis or Pectoralis major), so the possibility of a dissociation between one of the attachments and its intended skeletal target, as proposed in Bunnar et al., needs to be explored [56]. Freed from the initial association with a “typical” target, Wolpert’s characterization of flexibility in acquiring final skeletal attachments must be considered [50].

For the M sternalis, a problem in this part of the process should be evident in relative consistency in the appearance of the muscle, but with variations in the points of attachment. However, at least one of the attachments should be consistent with the expected locations of regular, named muscles.

## 4. Discussion

In the gross anatomy lab, students occasionally encounter atypical arrangements of skeletal muscle. Since the donors in our labs are usually older adults, it is easiest to observe gross anatomic features—size, shape, location, attachments—which only go part of the way in helping them to understand the appearance of the variant and its relationship to other anatomic structures.

Although it is often described as a “rare” variant [6,7], the appearance of M sternalis was documented as early as the 17th century [14], and its identification as a known anatomic variant is common through the early 20th century [10,13,20,21,22,23,24,25,26,27,28,29,30], after which it tends to appear only chiefly in case reports of unusual anatomic features. This may be because M sternalis appears to be of little clinical significance except for those who might encounter it in medical imaging or surgical professions [11,12,13,28,29].

The consensus on the muscle’s anatomic relations tends to shift, but current opinion seems mostly split between the Pectoralis major and the Rectus abdominis as potential sources for this muscle, based on its location, attachments, and innervation. The most disagreement seems to center on the innervation of this variant when it appears [10,13,20,21,22,23,24,25,26,27,28,29,30].

Phylogenetic analysis (Figure 4) shows that M sternalis is unlikely to be an “atavism” (see Table 1) failing most of the criteria proposed by Zanni and Opitz [35]. Since M sternalis usually appears in the absence of underlying skeletal or other connective-tissue abnormalities as it did in our lab, its presence is more likely due to local environmental influences on muscle development [6,20]. However, the wide variety of appearances of this variant does not indicate a single, consistent developmental driver of its presence, such as one might expect in known developmental disorders, such as Poland syndrome (https://omim.org/entry/173800) or various chromosomal mutations in which muscle development progresses atypically [8,58].

When confronted by such atypical skeletal muscles in gross anatomic dissection, there is an opportunity for enhancing learning with a deeper understanding of the structure and function of the human body. In the case of M sternalis, the first two essential questions—“What is it?” and “What is its function?”—are relatively easy to answer. The third—"Where did it come from?”—is the most challenging. To answer these questions, we explored the literature presented here to gather relevant information on the function, development, and evolutionary relationships of this muscle and its homologs. The information that we gathered can be evaluated using a heuristic model known as a “decision tree” to guide students through their explorations of atypical morphology. The goal is to make explicit the nature of the information they need to pass each decision node and to understand the nature of any anatomic variant that they encounter. It is not meant to lead them to a predetermined “correct” answer, and the conclusions could vary depending on the nature and quality of the resources that students have available to complete their background research.

**Figure 5:**
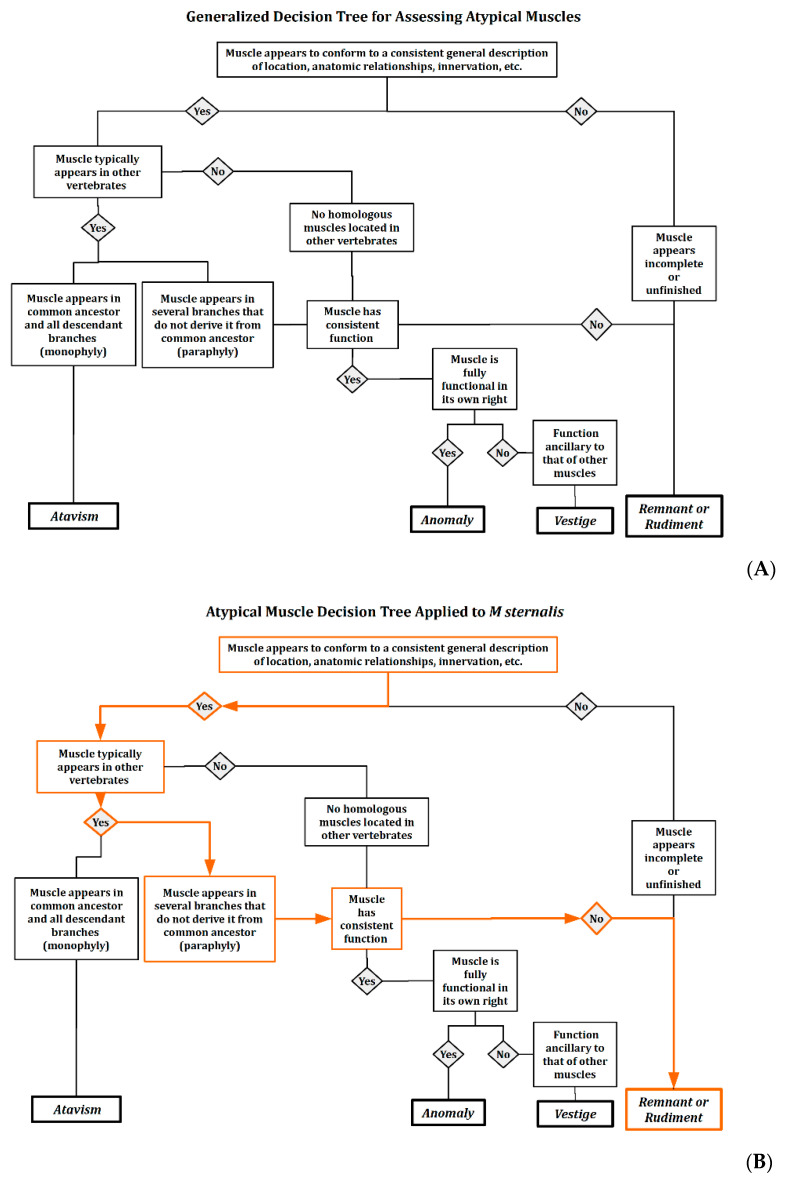
Decision Tree for assessing the likely sources of anatomic variations and areas for further inquiry. (**A**) Generalized form of decision tree showing nodes and links among pathways; (**B**) Application of decision tree to this particular example of M sternalis showing the realized decision pathway in orange.

In this case, we formulated the “decision tree” as a graphic organizer, though there are other ways in which the process could be visualized and engaged in by students. Figure 5A shows the generalized decision tree that can be applied to any muscle with an atypical appearance.

For this example, aligning their anatomic findings for function, evolution, and development, students can use the criteria in the boxes to assess their inferences about the source of the M sternalis variants. As they pass each node in the decision tree, they will either (a) reach a decision as to the most likely type of the muscle variant; or (b) identify the areas in which more investigation is needed before making a final determination. For example, we discovered through the process the importance of the careful identification and preservation of neurovascular supply for unusual muscle variants. In future dissections―even in the absence of M sternalis―we learned the value of carefully examining the innervation of cutaneous and subcutaneous tissues before proceeding to deeper layers.

Figure 5B shows the application of the decision tree to the problem of the M sternalis in our lab. The pathway through the decision tree is highlighted in orange. With the data available to us, we concluded that this example of M sternalis most likely represents a remnant or a rudimentary form of some muscle, because there is no consistent function; even though the muscle appears to be well formed in our example, its morphology is not consistent across all the examples in the literature.

In our lab, the muscle in question was well formed with well-developed and firm attachments. However, it is clear to us that the presentation in our lab was only one of the variants known for M sternalis. Had we been presented with one of the other variants, we might have followed a different path through the decision tree, although most of the variants we see in the literature would still lead us to the same final conclusion.

The decision tree serves as a heuristic: a template for the process of inferring the nature of anatomic variants that present in the dissection lab. It allows students to view different aspects of nature of anatomic features and to focus any further research or discussion of alternative findings on specific issues related to any feature’s function, evolutionary history, and development. The result is that students will have a more comprehensive appreciation for skeletal muscle structure and function that will enhance their understanding not only of atypical anatomic features, but of normal anatomy as well, and if they should palpate an unusual or unexpected muscle mass in their clinical practices, they will have a process for investigating and understanding the musculoskeletal variant that they have encountered.

## Figures and Tables

**Table 1:** Explanations for skeletal muscle variants.

Label	Description	Example
Vestige	A rarely used muscle often appearing in a variable form, but with regular attachments, innervation, and vascularization. Any associated function is usually redundant with functions performed regularly by other muscles.	M palmaris longus or M plantaris; both are variably present and add little to the function of other muscles that share their attachments; their absence likewise has little or no impact on function.
Anomaly	A muscle that normally appears in humans, but its development is modified from its normal course.	Hypertrophy of M abductor digiti minimi or M quadratus plantae [7].
Rudimentary	A muscle that would normally appear in humans, but its development has been interrupted.	Embryonic muscles that fail to regress and lead to the persistence of a whole muscle or of small muscle slips as in M pectorodorsalis in individuals with trisomy 21 [7].
Atavism	A muscle that is normally present in our evolutionary ancestors and typically missing in humans, but which re-appears in humans.	Chondroepitrochlearis is located along the inferior surface of the Pectoralis major and inserts on the medial aspect of the intermuscular septum and medial epicondyle of the humerus. Diagnosed as a remnant of Panniculus carnosus [32] or as a derivative of the pectoralis group [33].

**Table 2:** Template for describing muscles.

Student Prompts	Example: Pectoralis Minor
Name this muscle	Pectoralis minor
Principal attachments	Anterior surfaces ribs 3‒5; coracoid process of scapula
Muscle shape	Convergent as a whole, but individual slips can be parallel
Joint moved (for each joint that is located between the principal attachments)	Scapulothoracic and sternoclavicular
Functional characteristics of joint (plane, pivot, gliding, etc.)	Gliding (S-T) and sellar (S-C)
Type of movements allowed by joint	Depression/elevation; protraction/retraction; rotation
Planes of movements allowed by joint	Frontal and transverse
Directional relationship between proximal and distal attachments with respect to joint; for example, inferolateral to superomedial, etc.	Inferoanteromedial to superoposterolateral
Muscle’s “Line of Pull” (orientation of main axis of muscle action relative to the segments connected by the joints) in the anterior–posterior and medial–lateral axes; for example, anterolateral or anteromedial, etc.	Inferoanteromedial
These features of the muscle combined with the movement allowed at the joints, causes this change in position	Depression, abduction, and medial rotation of scapula; downward rotation of glenoid fossa (also clavicular depression and protraction of shoulder girdle)
Of this segment of the body	Scapula, clavicle, shoulder girdle collectively
In this (these) plane(s)	Depression and rotation: frontal; protraction: transverse
Innervation	Pectoral nn (C6‒C8)

**Table 3:** Appearance of muscles similar to *M sternalis* in humans and other species.

Taxa	Description	Name	Appearance	Source
Urodeles	Lengthwise along ventral body wall between girdles	Rectus abdominis “group”	Not specified	[37,38]
Ruminants	Thin muscle from first rib to sternum and costal cartilages 3‒5	Rectus thoracis	All species, though reduced in sheep and goats	[39]
Equines	Thin muscle from first rib to costal cartilage 4 and aponeurosis of Rectus abdominis	Rectus thoracis	Domestic horses	[39]
Hominids	Strap-like muscle from sternoclavicular joint or first rib variably to cranial attachment of Rectus abdominis	Sternalis	Variable appearance in *Homo* and *Hylobates syndactylus*	[3]
Baboons	Extension of Rectus abdominis aponeurosis to attach to manubrium	Rectus abdominis	Varies by species in genus *Papio*	[40]
	Aponeurosis with fleshy stratum	Rectus thoracis	*Papio papio*	[41]
Macaques	Aponeurosis fused with Rectus abdominis	Sternocostalis, Rectus thoracis, Rectus sternalis	*Macaca irus,* variably in *M sylvana*	[42]
	Extension of Rectus abdominis aponeurosis to attach to manubrium	Rectus abdominis	*Macaca mulatta*	[43]
Langurs and Tarsiers	Rectus abdominis extends to 1st costal cartilage	Rectus abdominis (abdominothoracic musculature)	*Semnopithecus,* occasionally in *Tarsius*	[44]
New World Monkeys	Rectus abdominis extends variably high into chest	Rectus abdominis	Various Hapalidae and Callimiconidae; genus *Cebus*	[45,46]
